# Supplementary material for: Paediatric chronic fatigue syndrome patients’ and parents’ perceptions of recovery
Source: BMJ Paediatr Open. 2019 Dec 2;3(1):e000525. doi: 10.1136/bmjpo-2019-000525 (PMC7245384; doi:10.1136/bmjpo-2019-000525)
Supplement: Supplementary data [file bmjpo-2019-000525supp001.pdf]

Appendices

Appendix 1: Topic guide

Introduction

1. **How does having CFS/ME affect your life?**

Prompts: Can you tell me more about X, what is it about X, can you describe that…

Recovery

2. **How do you know when you are feeling worse?**
3. **How do you know when you are feeling better?**
4. **What things if any stay the same whether you are better or worse?**

5. **How would you know if you were completely better again?**

Prompts: What would need to improve for you to say that you are better, by how much?

What would you like to do that you are unable to now?

What things can you cope with if they don't improve/ return to normal?

Minimal Clinically Important Difference (MCID)

MCID= change in the measure that a child thinks makes a difference to their lives.

Get YP to fill out SF36 for how they feel now. Take a red pen and get the YP to mark the SMALLEST AMOUNT the responses would have to change to make a difference to them.

6. **What would be the SMALLEST AMOUNT these would have to change by for you to feel like treatment has made a difference to you?**

Prompts: How many responses would have to improve, by how much…

Outcome Domains

Activity: Present YP with outcome domain cards. Add any domains to the blank cards the YP may have spontaneously brought up. Get YP to select the areas most affected by CFS/ME and rank them in order of importance as to what is most important to improve.

Top 5 domains- explore dimensions of domains (frequency/ severity/ duration/ satisfaction)

Prompts e.g. what symptoms bother you most, what is important about school.

[Take a photo of order].

7. **Pick out the top issues/areas of your life most affected by CFS/ME (upto 10).**
8. **Imagine you were able to improve these areas, rank these in order of what you feel is most important to improve, put the areas at the top you would most like to improve.**

    Tiredness

    Symptoms (pain, headaches, feeling sick, brain fog)

    Sleep problems

    Daily activities (getting up, getting dressed, going out)

    Payback & crashing (tired after activity)

    Fluctuation (changing symptoms- good day vs. bad day)

    School (attendance, concentrating, keeping up with work)

    Activities & hobbies (sports, clubs)

    Spending time with friends

    Family activities

    Mood (feeling down, worrying)

    How you feel about yourself (confidence, personality)

    Your future (GCSEs, college, jobs)

    Independence (doing things without your parents)

    Seeing your boyfriend/girlfriend

9. **Why have you ranked them in that order?**
    Top 5 domains- explore dimensions of domains (frequency/ severity/ duration/ satisfaction)
    - Prompts e.g. what symptoms bother you most, what is important about school.
10. **How might your answer have been different a year ago? [Pick the cards].**
    - Why were they important?
    - In what ways has it changed/ improved?

<u>**Ending question**</u>

11. **Is there anything else about having CFS/ME that you feel is important to you?**

Appendix 2: Additional supporting Quotes

**Descriptions of recovery**	
(Child-age-12)	"I don't really know, because I've been like this for so long that I don't know when I will be better, because it's not like when I am better it's like

	I don't know if I am, because this now is normal for me."
(Parent)	"…I don't think I can honestly answer that. Cos he's been ill for so long."
(Child-age-17)	"I think if I was 100% better than I would be able to do everything that I could do before I actually got it, which was quite a lot."
(Parent)	"…You want to get her to do what other teenagers are doing…"
(Parent)	"so just resuming a bit of normality really. Behaving like a teenager, going into town, you know, seeing friends, that kind of thing."
(Parent)	"…that is one of the most ridiculous questions in the sense that of course she'd know. If you have flu then you know when you're better…"
**Returning to socialising, education, and activities**	
(Child-age-17)	"I wouldn't be missing any days off like college..."
(Child-age-14)	"I'd be able to do more school work, which'd be nice."
(Child-age-17)	"I think a big thing is getting back into exercise, because obviously that was always quite a big part of what I used to....."
(Parent)	"…it would be just being able to yeah, go to school, do a full day…"
(Parent)	"If she started doing some physical exercise…"
(Child-age-15)	"more swimming and go to the gym"
**Recovery as an individualised process**	
(Child-age-17)	"... I would just rather be back how I was, but obviously if there could be like an improvement on one element of it could go, that would be really great. But obviously I would rather go back to how it was before…"
(Child-age-17)	"…I mean I'm the sort of person to just get on with things and achieve what I want to achieve regardless of how I feel, like I don't care if I've got a headache I will go and study and just get the work done. But it's just when it's a combination of being tired, headache, feeling sick, like achy, then it's just like exhausting."
(Parent)	"I don't want her to have any symptoms"
**The absence of 'Pay-Back'**	
(Child-age-13)	"I could do things and I wouldn't get backache"
(Parent)	"…to me he won't be fully recovered until he is like he says walking backwards and forwards to his school, at school all day and coming home, having a snack or whatever, mucking around with his brother,

	playing on the computer, and not having the payback afterwards really”
**Freedom and Spontaneity**	
(Child-age-17)	“... and just kind of maybe not having to plan everything quite as much like, because often you know like if we finish at lunchtime, “Oh do you want to go out for lunch?” I’m kind of like, “No I need to go home.” But I think yeah just being able to just be like yeah okay I’ll come with you, like kind of yeah things like that…”
(Child-age-13)	“…I'd be able to do what I like and do as much as I like and not have to go to bed early, get up early.”
(Parent)	“…..I think she’d be able to stay up later at night…..”
(Child-age-12)	“walking, erm, walk around for a long amount of time, erm, stand up for a long amount of time”
**Changes in Mood and Motivation**	
(Parent)	“...he would be able to just get up in the morning, and do a full day without any change to his personality or with him feeling anxious…”
