# Supplementary material for: Paediatric chronic fatigue syndrome patients’ and parents’ perceptions of recovery
Source: BMJ Paediatr Open. 2019 Dec 2;3(1):e000525. doi: 10.1136/bmjpo-2019-000525 (PMC7245384; doi:10.1136/bmjpo-2019-000525)
Supplement: Author's manuscript [file bmjpo-2019-000525.draft_revisions.pdf]

BMJ Paediatrics Open is committed to open peer review. As part of this commitment we make the peer review history of every article we publish publicly available.

When an article is published we post the peer reviewers' comments and the authors' responses online. We also post the versions of the paper that were used during peer review. These are the versions that the peer review comments apply to.

The versions of the paper that follow are the versions that were submitted during the peer review process. They are not the versions of record or the final published versions. They should not be cited or distributed as the published version of this manuscript.

BMJ Paediatrics Open is an open access journal and the full, final, typeset and author-corrected version of record of the manuscript is available on our site with no access controls, subscription charges or pay-per-view fees (http://bmjpaedsopen.bmj.com).

If you have any questions on BMJ Paediatrics Open's open peer review process please email info.bmjpo@bmj.com

BMJ Paediatrics Open

PAEDIATRIC CHRONIC FATIGUE SYNDROME PATIENTS' AND PARENTS' PERCEPTIONS OF RECOVERY

Journal:	*BMJ Paediatrics Open*
Manuscript ID	bmjpo-2019-000525
Article Type:	Original article
Date Submitted by the Author:	18-May-2019
Complete List of Authors:	Harland, Matthew; University of Bristol, Centre for Academic Child Health<br>Parslow, Roxanne; University of Bristol, Centre for Academic Child Health<br>Anderson, Nina; University of Bristol, Centre for Academic Child Health<br>Byrne, Danielle; University of Bristol, Centre for Academic Child Health<br>Crawley, Esther; University of Bristol, Centre for Academic Child Health
Keywords:	Chronic Fatigue Syndrome, Qualitative research, Patient perspective

SCHOLARONE™
Manuscripts

PAEDIATRIC CHRONIC FATIGUE SYNDROME PATIENTS' AND PARENTS' PERCEPTIONS OF RECOVERY

AUTHOR LIST

Matthew Harland1, Roxanne, M. Parslow1, Nina Anderson1, Danielle Byrne1, Esther Crawley1.

1. Centre for Academic Child Health, Bristol Medical School, University of Bristol, Bristol, England

Corresponding author: Matthew Harland
Centre for Academic Child Health
University of Bristol
Office 1.14
1-5 Whiteladies Road
Bristol
BS8 1NU

+44 (0) 117 42 83073

Email: mh14085@my.bristol.ac.uk

Key words: Chronic Fatigue Syndrome/Myalgic Encephalomyelitis, Children, Recovery, definitions.

Word count: 2577

(*Excluding title page, abstract, references, figures and tables)*

Confidential: For Review Only

ABSTRACT

Objectives

Chronic Fatigue Syndrome/Myalgic Encephalomyelitis (CFS/ME) is common in children and adolescents; however, little is known about how we should define recovery. This study aims to explore perceptions of recovery held by paediatric CFS/ME patients and their parents.

Methods

Children with CFS/ME and their parents were recruited through a single specialist paediatric CFS/ME service. Data was collected through semi structured interviews with children and parents. The interview questions explored how participants would know if they/their child had recovered from CFS/ME. Thematic analysis was used to identify patterns within the data.

Results

Twenty-one children with CFS/ME, twenty mothers and two fathers were interviewed. Some children found it hard to define recovery as the illness had become a 'new normal'. Others thought recovery would indicate returning to pre-morbid levels of activity or achieving the same activity level as peers (socialising, education, and leisure activities). Increased flexibility in routines and the absence of payback after activities were important. The interviews highlighted the concept of recovery as highly individual with wide variation in symptoms experienced, type and level of activity that would signify recovery. Parents describe how changes in mood and motivation would signify their child's recovery, but children did not reflect on this.

Confidential: For Review Only

Conclusion

Some parents and children struggle to define what would constitute complete recovery. However, signs of recovery were more easily identifiable. Definitions of recovery went far beyond symptom reduction and were focussed towards rebuilding lives.

Confidential: For Review Only

INTRODUCTION

Chronic Fatigue Syndrome/Myalgic Encephalomyelitis (CFS/ME) is common in children and adolescents, with a prevalence of 0.6-2.4% (1-6). It is characterised by severe fatigue and additional symptoms which get worse on exertion including: sleep disturbance, headaches, nausea, musculoskeletal pain, dizziness, and cognitive dysfunction (7). Children with CFS/ME have a reduced quality of life, low school attendance and many develop anxiety and depression (8, 9).

It is difficult to define recovery in CFS/ME as the condition affects multiple dimensions and improvements can be measured in physical, psychosocial functioning, and daily activities making it problematic to define “normalisation” of symptoms and function (10, 11). Little is known about how to define recovery in children with CFS/ME; they have described important areas of their lives impacted by the condition including: symptoms, physical activity, social participation (including school), and emotional well-being (12). This paper aims to explore how children with CFS/ME and their parents define recovery from the condition.

Confidential: For Review Only

METHOD

Participants

This was part of a larger qualitative study exploring how recovery should be measured in paediatric CFS/ME, and what improvements in fatigue and disability are important to young people and their parents. Children with CFS/ME and their parents were recruited in out-patient clinics provided by a specialist paediatric CFS/ME service. Children were eligible if they were diagnosed with CFS/ME (7), mild to moderately affected (not housebound), aged between 12- 17.99 years and able to understand the patient information sheets. Maximum variation purposive sampling (13) was undertaken to ensure a range of children (age, gender, and disease severity) participated. Children and parents provided the appropriate consent/assent prior to interview.

Data collection

Semi structured interviews were undertaken by three researchers (RMP, NA, DB) between December 2014 and February 2015. Participants were offered interviews at home or on hospital premises to reduce travel (14). We aimed to interview children and parents separately (15) but families were given the option of being together if they preferred.

A flexible open-ended interview guide was developed with input from a young person's advisory group. The topic guide, included questions on recovery, relevant outcomes to measure, the amount of change considered important (the minimal clinically important difference) and a section on recovery. This section included questions on how participants knew when they were feeling worse, better, and how they would know if they had recovered ***(extract, Appendix 1***).

https://mc.manuscriptcentral.com/bmjpo

Confidential: For Review Only

Interviews continued until data saturation was achieved (16).

Data analysis

All interviews were recorded on an encrypted digital audio recorder and transcribed verbatim. Transcripts were checked for accuracy, anonymised and uploaded to NVIVO 10 (17) to provide an audit trail for data analysis. Thematic analysis (18) was used to identify patterns (themes) within the data in an iterative process. Codes were initially developed "in vivo" inductively from participants own language in order to represent concepts important to families with CFS/ME. Codes were then added, merged, or split as analysis progressed to reflect emerging dimensions in the data (19). This allowed higher-level recurring themes to be developed and the data was checked between participants to see if matched the overall coding. A summary was then written with illustrative quotes to highlight the overall themes and differing perspectives (20).

Quality assurance

The first three interview transcripts were reviewed for interviewer skill (following the topic guide, picking up on cues, avoid leading questions) (21) and discussed in a meeting with feedback from a senior researcher (EC) (EC, RP, NA, DB). The interview transcripts were double coded and themes discussed between researchers (MH, RP) to compare coding, highlight any significant differences to improve the trustworthiness of analyses and enhance interpretation (22-24). Coding was found to be very similar, at times alternative wording was used for the same codes.

Patient and public involvement

A young person's advisory group comprising secondary school children was involved in the development of the interview topic guide during the design stages of this study.

Confidential: For Review Only

RESULTS

Participants

Twenty-one children with CFS/ME were interviewed, 16 (76%) were female and 5 (24%) were male, mean age was 14.4 years and ages ranged from 12 to 17 years old. Seventeen (81%) children had mild CFS/ME and 4 (19%) had moderate CFS/ME. Twenty mothers and two fathers were interviewed. Most children and parents were interviewed separately but in 4/21 interviews a parent was present in the room during the child's interview. Five families who had initially expressed interest in participating when being contacted by the researcher did not participate (two were too busy and three children were too ill).

Difficulty defining recovery

Some children found it difficult to describe how they would know if they had recovered. Living with the condition had become a 'new normal' for them and as they had had CFS/ME for so long, children and their parents couldn't imagine recovery. Due to the fluctuating nature of the illness one child felt that even if she had recovered she would worry about relapsing:

> "I don't really think I would know. I think like now it's constantly at the back of mind like whether or not like it will knock me again..." (Child-age-17)

Parents and children both had a tendency to report signs of improvement rather than signs of complete recovery. Some children, but more parents, stated that they would have no difficulty in knowing when they/their child had recovered as they would be 'back-to-normal' engaging in pre-morbid levels of functioning or the same activity as their peers ***(additional***

Confidential: For Review Only

quotes supporting each theme can be found in Appendix 2):

"I think we would know because she'd be back to how she was before sort of the illness …You know, there's that reference point in time that you can go back to." (Parent)

Returning to socialising, education, and activities

All children reported that recovery would be indicated by a return to activities that were particularly important to them: socialising with friends, attending school/college regularly, physical activities, and cognitive activities. Often, children described school attendance as important for social not educational reasons. The same categories were also reported as important indicators of recovery by parents.

"Probably school, I'd say – - 'cause I think, if I get back into school then I can like, get in touch with more people" (Child-age-13)

Recovery as an individualised process

There was wide variation in types of activities that children and parents described as indicating recovery. For children, these activities included cooking, playing instruments, singing, and creating artwork. For parents, these included activities with the family, travel, holidays, and dog-walks. For one family, both the parent and child described increased mobility to be the most significant indicator of recovery as the child was reliant on a wheelchair.

"...knows what she wants to do, if she could do it she would, and her main aim is now to probably try and get round the shops without her wheelchair…" (Parent)

In contrast, other parents described a much higher level of activity to signify recovery:

Confidential: For Review Only

"…on the days when gymnastics was on, she exercised every single day, and that's how I know when I think when she's better it's when she starts physical activity again." (Parent)

The idea that recovery is highly individualised was also evident in the descriptions of symptoms children could tolerate as part of their recovery. Some parents and children stated that no symptoms would be acceptable in recovery. However, some children felt that they would be able to consider themselves recovered even if they still had some symptoms. Children had different views over whether tiredness was bearable or not and which symptoms were tolerable.

"I'd be able to live with my headaches." (Child-age-14)

"…the headaches because they, erm, they just stop her being able to think…" (Parent)

The absence of 'Payback'

As well as returning to premorbid functioning, children and parents felt that increasing activities should not result in payback (the increase in symptoms following activity) in order for them to be considered recovered.

"I think I'd know if like, I went out and did something, I didn't get payback for it, I think that one, I'd know that I was like really starting to recover" (Child-age-13)

Freedom and Spontaneity

Children had become used to planning their activity in a way that facilitated pacing and reduced payback. They felt that life had become regimented and that they were unable to spontaneously choose to do an activity/continue without a break. The absence of this rigidity of routine was an important indicator of recovery.

Confidential: For Review Only

"You'd know when she's recovered because she'd be back to normal, because she'd be able to do things, and not just do them but do them without thinking about how much energy she was using." (Parent)

For some parents and children, recovery also incorporated the idea that they would be able to have more flexibility in their sleep routines, without experiencing payback. In contrast, some parents felt that the monitoring of activity would be acceptable in recovery to help limit tiredness.

Changes in Mood and Motivation

Parents expressed that recovery would be indicated by a change in their child's mood. For some, this would be a reduction in anxiety, and for others, it would be increased positive mood and more engagement with others:

"Well if she was completely better she would be lively again, bubbly again, joking and laughing again, singing again, but all the time, that's how she would be." (Parent)

Often, parents described that their children would be more motivated when they had recovered. This position was not reflected in the responses of children who often appeared motivated but cautious and limited by their symptoms.

"So if he was better you'd think he'd just be motivated to get up and be physically able" (Parent)

Some parents recognised that changes in personality and preference in activities in their children may be due to factors other than CFS/ME.

"Yeah, she's hit puberty as well so, you know, I think a lot of the sort of withdrawing kind of is a teenage thing as well, like wanting to be on her own." (Parent)

Confidential: For Review Only

DISCUSSION

This is the first paper to explore what children with CFS/ME feel about recovery. There is a struggle to define what would constitute complete recovery as CFS/ME has become a 'new normal'. Previous levels of activity prior to becoming ill or keeping up with peers were key markers. Descriptions of recovery went far beyond symptom reduction and focussed on rebuilding lives which incorporated valued activities in multiple areas of life (socialising, education, and activities). The ability to be flexible and spontaneous without suffering payback was an important theme. However, the concept of recovery was highly individual; there was heterogeneity in the activities children wanted to return to as well as the extent to which different symptoms would be acceptable in recovery. There are differences between child and parent definitions of recovery in CFS/ME, particularly surrounding the role of mood.

Strengths and weaknesses

This is a large qualitative study including children (n=21) and parents (n=22) which reached data saturation (16). As we only interviewed children who were mild/moderately affected, the results are not generalizable to those who are severely affected or younger than 12 years old. Most children chose to be interviewed without their parents, allowing the exploration of differences between child and parent. In the four interviews where parents were present, the child may not have been able to speak freely (25). Children were recruited from one specialist service, therefore, the results may not be generalizable to the whole of the UK. Fewer males were recruited (5) and fathers (2) therefore limiting the findings in these sub groups; however, this is consistent with previous research (26).

Confidential: For Review Only

Results in context with previous literature

Children and parents in this study struggled to define recovery from CFS/ME. This is consistent with the recovery literature in adults with CFS/ME (27). Trouble defining recovery appeared to relate to uncertainty surrounding self-identity; living with the illness had become a ‘new normal’. This reflects the biographical disruption reported in previous research (28). For a lot of children, the focus of recovery had moved away from symptom reduction and towards re-building their lives and identity; a finding that has been reported in the adult CFS/ME population (29).

Parents and children varied in their criteria for recovery, corresponding with the variety of definitions used in the literature (11, 27). Parents suggested a return to functioning at pre-morbid levels or at the level of peers to signify recovery. This is consistent with attempts to characterise recovery as being a return to population norms, equivalent to physician held views (10). Previous research on adult patient’s recovery stories in CFS/ME drew on comparisons between themselves currently and themselves prior to the illness or peers (30). However, ‘pre-morbid’ as a marker is problematic as children in this study varied in their levels of physical function and participation prior to becoming ill, which may change over the course of the illness and memory of pre-morbid levels falls short of an objective measure (31). Similarly, suggestions that recovery would be signified by an increase in activity levels to match those of the children’s peers, reflects the research in the adult CFS/ME population relating to the normalisation of function. However, this is difficult to define for the same reasons as in the adult population (wide variation for different ages and the presence of co-morbid health problems) (11).

https://mc.manuscriptcentral.com/bmjpo

Confidential: For Review Only

This study identified key areas of life in which recovery would be recognisable (education, socialising, and physical activity). The same areas have been identified in previous research into the development of a patient reported outcome measure (PROM) for use In this population (12). However, this paper highlights that the specific details of what recovery means, is different for each individual. The ability to be spontaneous without suffering payback was an important marker for recovery in this study, further research is needed to understand how children describe and understand payback.

Confidential: For Review Only

IMPLICATIONS

This research highlights the individuality of defining recovery in children with CFS/ME. This suggests that it is vital to involve children in developing any new patient reported outcome measure for paediatric CFS/ME (15). Use of a patient generated index in which patients select the most important areas of their lives and rate their health in those areas may be more appropriate (32). Research studies should consider using questions where patients self-define recovery (e.g. “I have recovered”, or “I have nearly recovered”). Clinicians should be aware of the differences between their own goals for recovery and those of parents and children.

Confidential: For Review Only

ACKNOWLEDGEMENTS

We are grateful to all the children and parents who took part in this study.

FUNDING

This work was supported by a University of Bristol PhD Scholarship. EC was funded by the National Institute for Health Research (Senior Research Fellowship, SRF-2013-06-013). The views expressed in this publication are those of the authors and not necessarily those of the NHS, the National Institute for Health Research or the Department of Health.

COMPETING INTERESTS

All authors declare they have no financial or non-financial interests that may be relevant to the submitted work. EC was the medical advisor for the Association for Young people with ME (AYME) until 2017.

AUTHORS' CONTRIBUTIONS

EC designed the study. RP, NA and DB collected the data. RP and MH analysed the data. All authors contributed to the interpretation of results and to drafting this paper. All authors have read and approved the final version of the manuscript.

DATA SHARING

No additional data available.

ETHICS APPROVAL

Confidential: For Review Only

Full ethical approval was obtained from the NRES Committee North West (08/04/2014, ref 14/NW/0170). R&D approval was obtained from the RNHRD (20/06/2014, ref-RBB 427).

Confidential: For Review Only

What is already known on this topic
• Little is known about how to define recovery in the paediatric CFS/ME population.
**What this study adds**
• Children and their parents struggle to define what would constitute a complete recovery as CFS/ME has become a 'new normal'.
• There is wide variation in definitions of recovery between individuals.
• Recovery definitions go beyond symptom reduction and focus on returning to or achieving the same activity as peers, without payback and with flexibility in routine.

Confidential: For Review Only

References

1. Rimes KA, Goodman R, Hotopf M et al. Incidence, prognosis, and risk factors for fatigue and chronic fatigue syndrome in adolescents: a prospective community study. *Pediatrics*. 2007;119(3):e603-9.
2. Chalder T, Goodman R, Wessely S et al. Epidemiology of chronic fatigue syndrome and self reported myalgic encephalomyelitis in 5-15 year olds: cross sectional study. *Bmj*. 2003;327(7416):654-5.
3. Crawley EM, Emond AM, Sterne JA. Unidentified Chronic Fatigue Syndrome/myalgic encephalomyelitis (CFS/ME) is a major cause of school absence: surveillance outcomes from school-based clinics. *BMJ Open*. 2011;1(2):e000252.
4. Crawley E, Hughes R, Northstone K et al. Chronic disabling fatigue at age 13 and association with family adversity. *Pediatrics*. 2012.
5. Haines LC, Saidi G, Cooke RW. Prevalence of severe fatigue in primary care. *Arch Dis Child*. 2005;90(4):367-8.
6. Nijhof SL, Maijer K, Bleijenberg G et al. Adolescent chronic fatigue syndrome: prevalence, incidence, and morbidity. *Pediatrics*. 2011;127(5):e1169-75.
7. NICE NIfHaCE. Chronic fatigue syndrome/myalgic encephalomyelitis (or encephalopathy): Diagnosis and management of CFS/ME in adults and children: *National Institute for Clinical Excellence*; 2007.
8. Garralda ME, Rangel L. Impairment and coping in children and adolescents with chronic fatigue syndrome: a comparative study with other paediatric disorders. *Journal of Child Psychoogy andl Psychiatry*. 2004;45(3):543-52.
9. Crawley E, Sterne JA. Association between school absence and physical function in paediatric chronic fatigue syndrome/myalgic encephalopathy. Arch Dis Child. 2009;94(10):752-6.
10. Devendorf AR, Jackson CT, Sunnquist M et al. Defining and measuring recovery from myalgic encephalomyelitis and chronic fatigue syndrome: the physician perspective. *Disabil Rehabil*. 2017:1-8.
11. Matthees A. Assessment of recovery status in chronic fatigue syndrome using normative data. *Qual Life Res*. 2015;24(4):905-7.
12. Parslow R, Patel A, Beasant L et al. What matters to children with CFS/ME? A conceptual model as the first stage in developing a PROM. *Arch Dis Child* 2015.
13. Ritchie J, Lewis J, Nicholls CM et al. Qualitative research practice: *A guide for social science students and researchers*: Sage; 2013.
14. Crawley E, Hunt L, Stallard P. Anxiety in children with CFS/ME. *Eur Child Adolesc Psychiatry*. 2009;18(11):683-9.
15. Matza LS, Patrick DL, Riley AW et al. Pediatric patient-reported outcome instruments for research to support medical product labeling: report of the ISPOR PRO good research practices for the assessment of children and adolescents task force. *Value Health*. 2013;16(4):461-79.
16. Guest G, Bunce A, Johnson L. How many interviews are enough? An experiment with data saturation and variability. *Field methods*. 2006;18(1):59-82.
17. Castleberry A. NVivo 10 [software program]. Version 10. QSR International; 2012. AJPE; 2014.
18. Braun V, Clarke V. Using thematic analysis in psychology. *Research in Psychology*. 2006;3(2):77–101.
19. Pope C, Mays N. *Qualitative Research in Health care*: Blackwell Publishing Ltd; 2006.
20. Pope C, Ziebland S, Mays N. Qualitative research in health care: analysing qualitative data. *BMJ*. 2000;320(7227):114.
21. Britten N. Qualitative interviews in medical research. *Bmj*. 1995;311(6999):251-3.

Confidential: For Review Only

22. Johnson RB. Examining the validity structure of qualitative research. *Education*. 1997;118(2):282.
23. Mays N, Pope C. Assessing quality in qualitative research. BMJ. 2000;320(7226):50-2.
24. Pope C, Mays N. Qualitative research: rigour and qualitative research. *BMJ*. 1995;311:109-12.
25. Grills AE, Ollendick TH. Issues in parent-child agreement: the case of structured diagnostic interviews. *Clin Child Fam Psychol Rev*. 2002;5(1):57-83.
26. Missen A, Hollingworth W, Eaton N et al. The financial and psychological impacts on mothers of children with chronic fatigue syndrome (CFS/ME). *Child Care Health Dev*. 2012;38(4):505-12.
27. Adamowicz JL, Caikauskaite I, Friedberg F. Defining recovery in chronic fatigue syndrome: a critical review. *Qual Life Res*. 2014;23(9):2407-16.
28. Parslow RM, Harris S, Broughton J et al. Childrens experiences of chronic fatigue syndrome/myalgic encephalomyelitis (CFS/ME): a systematic review and meta-ethnography of qualitative studies. *BMJ open*. 2017;7(1):e012633.
29. Whitehead L. Toward a trajectory of identity reconstruction in chronic fatigue syndrome/myalgic encephalomyelitis: A longitudinal qualitative study. *Int J Nurs Stud*. 2006;43(8):1023-31.
30. Brown B, Huszar K, Chapman R. 'Betwixt and between'; liminality in recovery stories from people with myalgic encephalomyelitis (ME) or chronic fatigue syndrome (CFS). *Sociol Health Illn*. 2017.
31. Brooks BL, Kadoura B, Turley B et al. Perception of recovery after pediatric mild traumatic brain injury is influenced by the "good old days" bias: tangible implications for clinical practice and outcomes research. *Arch Clin Neuropsychol*. 2013;29(2):186-93.
32. Ruta DA, Garratt AM, Leng M et al. A new approach to the measurement of quality of life: the Patient-Generated Index. *Medical Care*. 1994:1109-26.

Confidential: For Review Only

Appendices

Appendix 1: Extract of topic guide

Introduction

- How does having CFS/ME affect your life?

Recovery

- How do you know when you are feeling worse?
- How do you know when you are feeling better?
- How would you know if you were completely better/well again?

Closing question

- Is there anything else about having CFS/ME that you feel is important to you?

Confidential: For Review Only

Appendix 2: Additional supporting Quotes

**Descriptions of recovery**	
(Child-age-12)	"I don't really know, because I've been like this for so long that I don't know when I will be better, because it's not like when I am better it's like I don't know if I am, because this now is normal for me."
(Parent)	"…I don't think I can honestly answer that. Cos he's been ill for so long."
(Child-age-17)	"I think if I was 100% better than I would be able to do everything that I could do before I actually got it, which was quite a lot."
(Parent)	"…You want to get her to do what other teenagers are doing…"
(Parent)	"so just resuming a bit of normality really. Behaving like a teenager, going into town, you know, seeing friends, that kind of thing."
(Parent)	"…that is one of the most ridiculous questions in the sense that of course she'd know. If you have flu then you know when you're better…"
**Returning to socialising, education, and activities**	
(Child-age-17)	"I wouldn't be missing any days off like college..."
(Child-age-14)	"I'd be able to do more school work, which'd be nice."
(Child-age-17)	"I think a big thing is getting back into exercise, because obviously that was always quite a big part of what I used to....."
(Parent)	"…it would be just being able to yeah, go to school, do a full day…"
(Parent)	"If she started doing some physical exercise…"
(Child-age-15)	"more swimming and go to the gym"
**Recovery as an individualised process**	
(Child-age-17)	"... I would just rather be back how I was, but obviously if there could be like an improvement on one element of it could go, that would be really great. But obviously I would rather go back to how it was before…"
(Child-age-17)	"…I mean I'm the sort of person to just get on with things and achieve what I want to achieve regardless of how I feel, like I don't care if I've got a headache I will go and study and just get the work done. But it's just when it's a combination of being tired, headache, feeling sick, like achy, then it's just like exhausting."

Confidential: For Review Only

(Parent)	"I don't want her to have any symptoms"
**The absence of 'Pay-Back'**	
(Child-age-13)	"I could do things and I wouldn't get backache"
(Parent)	"…to me he won't be fully recovered until he is like he says walking backwards and forwards to his school, at school all day and coming home, having a snack or whatever, mucking around with his brother, playing on the computer, and not having the payback afterwards really"
**Freedom and Spontaneity**	
(Child-age-17)	"... and just kind of maybe not having to plan everything quite as much like, because often you know like if we finish at lunchtime, "Oh do you want to go out for lunch?" I'm kind of like, "No I need to go home." But I think yeah just being able to just be like yeah okay I'll come with you, like kind of yeah things like that…"
(Child-age-13)	"…I'd be able to do what I like and do as much as I like and not have to go to bed early, get up early."
(Parent)	"…..I think she'd be able to stay up later at night….."
(Child-age-12)	"walking, erm, walk around for a long amount of time, erm, stand up for a long amount of time"
**Changes in Mood and Motivation**	
(Parent)	"...he would be able to just get up in the morning, and do a full day without any change to his personality or with him feeling anxious…"

Confidential: For Review Only

BMJ Paediatrics Open

PAEDIATRIC CHRONIC FATIGUE SYNDROME PATIENTS' AND PARENTS' PERCEPTIONS OF RECOVERY

Journal:	*BMJ Paediatrics Open*
Manuscript ID	bmjpo-2019-000525.R1
Article Type:	Original research
Date Submitted by the Author:	09-Oct-2019

Complete List of Authors:	Harland, Matthew; University of Bristol, Centre for Academic Child Health<br>Parslow, Roxanne; University of Bristol, Centre for Academic Child Health<br>Anderson, Nina; University of Bristol, Centre for Academic Child Health<br>Byrne, Danielle; University of Bristol, Centre for Academic Child Health<br>Crawley, Esther; University of Bristol, Centre for Academic Child Health

Keywords:	Chronic Fatigue Syndrome, Qualitative research, Patient perspective

SCHOLARONE™
Manuscripts

PAEDIATRIC CHRONIC FATIGUE SYNDROME PATIENTS' AND PARENTS' PERCEPTIONS OF RECOVERY

AUTHOR LIST

Matthew Harland1, Roxanne, M. Parslow1, Nina Anderson1, Danielle Byrne1, Esther Crawley1.

1. Centre for Academic Child Health, Bristol Medical School, University of Bristol, Bristol, England

Corresponding author: Esther Crawley
Centre for Academic Child Health
University of Bristol
Office 1.14
1-5 Whiteladies Road
Bristol
BS8 1NU

+44 (0) 117 42 83073

Email: Esther.Crawley@bristol.ac.uk

Key words: Chronic Fatigue Syndrome/Myalgic Encephalomyelitis, Children, Recovery, definitions.

Word count: 2577

(*Excluding title page, abstract, references, figures and tables)*

https://mc.manuscriptcentral.com/bmjpo

Confidential: For Review Only

ABSTRACT

Objectives

Chronic Fatigue Syndrome/Myalgic Encephalomyelitis (CFS/ME) is common in children and adolescents; however, little is known about how we should define recovery. This study aims to explore perceptions of recovery held by paediatric CFS/ME patients and their parents.

Methods

Children with CFS/ME and their parents were recruited through a single specialist paediatric CFS/ME service. Data was collected through semi structured interviews with children and parents. The interview questions explored how participants would know if they/their child had recovered from CFS/ME. Thematic analysis was used to identify patterns within the data.

Results

Twenty-one children with CFS/ME, twenty mothers and two fathers were interviewed. Some children found it hard to define recovery as the illness had become a 'new normal'. Others thought recovery would indicate returning to pre-morbid levels of activity or achieving the same activity level as peers (socialising, education, and leisure activities). Increased flexibility in routines and the absence of payback after activities were important. The interviews highlighted the concept of recovery as highly individual with wide variation in symptoms experienced, type and level of activity that would signify recovery. Parents describe how changes in mood and motivation would signify their child's recovery, but children did not reflect on this.

Confidential: For Review Only

Conclusion

Some parents and children struggle to define what would constitute complete recovery. However, signs of recovery were more easily identifiable. Definitions of recovery went far beyond symptom reduction and were focussed towards rebuilding lives.

Confidential: For Review Only

INTRODUCTION

Chronic Fatigue Syndrome/Myalgic Encephalomyelitis (CFS/ME) is common in children and adolescents, with a prevalence of 0.6-2.4% (1-6). It is characterised by severe fatigue and additional symptoms which get worse on exertion including: sleep disturbance, headaches, nausea, musculoskeletal pain, dizziness, and cognitive dysfunction (7). Children with CFS/ME have a reduced quality of life, low school attendance and many develop anxiety and depression (8, 9).

It is difficult to define recovery in CFS/ME as the condition affects multiple dimensions including symptoms, physical activity, social participation (including school), and emotional well-being (10). Improvements can be therefore be measured in physical, psychosocial functioning, and daily activities making it problematic to define recovery in terms of either symptoms or function (11, 12). Some randomised controlled trials have defined recovery as improvement in multiple domains, others as a return to normal within one domain.(13) However, these decisions on defining recovery have been made by researchers and clinicians and not derived from children and young people with CFS/ME. This paper aims to explore how children with CFS/ME and their parents define recovery from the condition. This is important for future research including treatment trials and outcome studies in paediatric CFS/ME.

Confidential: For Review Only

METHOD

Study Design

We recruited participants and their parents/carers to a qualitative study with three research questions: 1. How should we define recovery (this paper); 2. Which outcomes are important for children with CFS/ME and their parents (under review) and 3. What is the minimally clinically important difference in fatigue and SF-36 physical function scale (14). Participants and parents/carers were asked about each of these three areas. The answers to our research question on recovery are reported in this paper.

Interviews were used as it was not possible to recruit to focus groups as patients were from a wide geographical area requiring long distance travel placing a possible burden on children with CFS/ME who experience fatigue and payback. Individual interviews allow more in-depth exploration and interviewing has been used successfully from preschool to high school children with a range of conditions (diabetes, asthma, arthritis and bowel disease (15-17)).

Participants

Children with CFS/ME and their parents were recruited in out-patient clinics provided by a specialist paediatric CFS/ME service. Children were eligible if they were diagnosed with CFS/ME (7), mild to moderately affected (not housebound), aged between 12- 17.99 years and able to understand the patient information sheets. Maximum variation purposive sampling (18) was undertaken to ensure a range of children (age, gender, and disease severity) participated. Children and parents provided the appropriate consent/assent prior

Confidential: For Review Only

to interview. We recruited newly diagnosed patients and patients that were receiving follow up so that a range of views could be included.

Data collection

Semi structured interviews were undertaken by three researchers (RMP, NA, DB) between December 2014 and February 2015. Participants were offered interviews at home or on hospital premises to reduce travel (19). We aimed to interview children and parents separately (20) but families were given the option of being together if they preferred. Interviews took between 15 and 42 minutes. After 20 minutes, the interviewer checked with the participant that they were happy to continue.

A flexible open-ended interview guide was developed with input from a young person's advisory group within a secondary school (7 female, 1 male) to ensure the questions were comprehensible and clear to school aged children. The topic guide, included questions on recovery, relevant outcomes to measure, the amount of change considered important (the minimal clinically important difference) and a section on recovery. This section included questions on how participants knew when they were feeling worse, better, and how they would know if they had recovered ***(Appendix 1)***.

Interviews continued until data saturation was achieved (21).

Data analysis

All interviews were recorded on an encrypted digital audio recorder and transcribed verbatim. Transcripts were checked for accuracy, anonymised and uploaded to NVIVO 10 (22) to provide an audit trail for data analysis. Thematic analysis (23) was used to identify

Confidential: For Review Only

patterns (themes) within the data in an iterative process. Codes were initially developed “in vivo” inductively from participants own language in order to represent concepts important to families with CFS/ME. Codes were then added, merged, or split as analysis progressed to reflect emerging dimensions in the data (24). This allowed higher-level recurring themes to be developed and the data was checked between participants to see if matched the overall coding. A summary was then written with illustrative quotes to highlight the overall themes and differing perspectives (25).

Quality assurance

The first three interview transcripts were reviewed for interviewer skill (following the topic guide, picking up on cues, avoid leading questions) (26) and discussed in a meeting with feedback from a senior researcher (EC) (EC, RP, NA, DB). The interview transcripts were double coded and themes discussed between researchers (MH, RP) to compare coding, highlight any significant differences to improve the trustworthiness of analyses and enhance interpretation (27-29). Coding was found to be very similar, at times alternative wording was used for the same codes.

Patient and public involvement

A young person’s advisory group comprising secondary school children was involved in the development of the interview topic guide during the design stages of this study.

Confidential: For Review Only

RESULTS

Participants

Twenty-one children with CFS/ME were interviewed, 16 (76%) were female and 5 (24%) were male, mean age was 14.4 years and ages ranged from 12 to 17 years old. Seventeen (81%) children had mild CFS/ME and 4 (19%) had moderate CFS/ME. Twenty mothers and two fathers were interviewed. Most children and parents were interviewed separately but in 4/21 interviews a parent was present in the room during the child's interview. Five families who had initially expressed interest in participating when being contacted by the researcher did not participate (two were too busy and three children were too ill).

Difficulty defining recovery

Some children found it difficult to describe how they would know if they had recovered. Living with the condition had become a 'new normal' for them and as they had had CFS/ME for so long, children and their parents couldn't imagine recovery. Due to the fluctuating nature of the illness one older child felt that even if she had recovered she would worry about relapsing:

> "I don't really think I would know. I think like now it's constantly at the back of mind like whether or not like it will knock me again..." (Child-age-17)

Parents and children both had a tendency to report signs of improvement rather than signs of complete recovery. Some children, but more parents, stated that they would have no difficulty in knowing when they/their child had recovered as they would be 'back-to-normal' engaging in pre-morbid levels of functioning or the same activity as their peers ***(additional***

Confidential: For Review Only

quotes supporting each theme can be found in Appendix 2):

"I think we would know because she'd be back to how she was before sort of the illness …You know, there's that reference point in time that you can go back to." (Parent)

Returning to socialising, education, and activities

All children reported that recovery would be indicated by a return to activities that were particularly important to them: socialising with friends, attending school/college regularly, and both physical "more swimming and go to the gym" and mental activities "I think I'd just have a bit better memory" (Child-age-15).. Often, children described school attendance as important for social not educational reasons. The same categories were also reported as important indicators of recovery by parents.

"Probably school, I'd say – - 'cause I think, if I get back into school then I can like, get in touch with more people" (Child-age-13)

Being able to return to activities but do them for longer was important "walking, erm, walk around for a long amount of time, erm, stand up for a long amount of time" (Child-age-12).

Recovery as an individualised process

There was wide variation in types of activities that children and parents described as indicating recovery. For children, these activities included cooking, playing instruments, singing, and creating artwork. The types of activities children referred to were relevant for their age. For example, 16-17 year olds talked about wanting to be able to be more independent and "walk to town", whereas younger children talked about "sleepovers" and playing with friends, "run around and play a lot".

"…and having more independence in that sense. Like most people my age will go out to the

Confidential: For Review Only

shops, or just walk around aimlessly, that seems to be popular, but like [laughs] I wish I could do that like. (Child-age-16)

"Erm, I miss my sports. Erm, ju- have, have sleepovers, erm, with my friends" (Child-age-12)

For parents, these included activities with the family, travel, holidays, and dog-walks. For one family, both the parent and child described increased mobility to be the most significant indicator of recovery as the child was reliant on a wheelchair.

"...knows what she wants to do, if she could do it she would, and her main aim is now to probably try and get round the shops without her wheelchair…" (Parent)

In contrast, other parents described a much higher levels of activity to signify recovery:

"…on the days when gymnastics was on, she exercised every single day, and that's how I know when I think when she's better it's when she starts physical activity again." (Parent)

The idea that recovery is highly individualised was also evident in the descriptions of symptoms children could tolerate as part of their recovery. Some parents and children stated that no symptoms would be acceptable in recovery. However, some children felt that they would be able to consider themselves recovered even if they still had some symptoms. Children had different views over whether tiredness was bearable or not and which symptoms were tolerable.

"I'd be able to live with my headaches." (Child-age-14)

"…the headaches because they, erm, they just stop her being able to think…" (Parent)

The absence of 'Payback'

https://mc.manuscriptcentral.com/bmjpo

Confidential: For Review Only

As well as returning to premorbid functioning, children and parents felt that increasing activities should not result in payback (the increase in symptoms following activity) in order for them to be considered recovered.

"I think I'd know if like, I went out and did something, I didn't get payback for it, I think that one, I'd know that I was like really starting to recover" (Child-age-13)

Freedom and Spontaneity

Older children talked about how they had become used to planning their activity in a way that facilitated pacing and reduced payback. They felt that life had become regimented and that they were unable to spontaneously choose to do an activity/continue without a break. The absence of this rigidity of routine was an important indicator of recovery.

"…like won't do as much during the day so that then I can go out in the evening. Yeah, I think that's like that what I mean by like planning, it's just so I can kind of plan my other activities like accordingly I suppose. (Child-age-17).

"You'd know when she's recovered because she'd be back to normal, because she'd be able to do things, and not just do them but do them without thinking about how much energy she was using." (Parent)

For some parents and children, recovery also incorporated the idea that they would be able to have more flexibility in their sleep routines, without experiencing payback. In contrast, some parents felt that the monitoring of activity would be acceptable in recovery to help limit tiredness.

Changes in Mood and Motivation

Confidential: For Review Only

Parents expressed that recovery would be indicated by a change in their child's mood. For some, this would be a reduction in anxiety, and for others, it would be increased positive mood and more engagement with others:

"Well if she was completely better she would be lively again, bubbly again, joking and laughing again, singing again, but all the time, that's how she would be." (Parent)

Often, parents described that their children would be more motivated when they had recovered. This position was not reflected in the responses of children who often appeared motivated but cautious and limited by their symptoms.

"So if he was better you'd think he'd just be motivated to get up and be physically able" (Parent)

Some parents recognised that changes in personality and preference in activities in their children may be due to factors other than CFS/ME.

"Yeah, she's hit puberty as well so, you know, I think a lot of the sort of withdrawing kind of is a teenage thing as well, like wanting to be on her own." (Parent)

Confidential: For Review Only

DISCUSSION

This is the first paper to explore what children with CFS/ME feel about recovery. There is a struggle to define what would constitute complete recovery as CFS/ME has become a 'new normal'. Previous levels of activity prior to becoming ill or keeping up with peers were key markers. Descriptions of recovery went far beyond symptom reduction and focussed on rebuilding lives which incorporated valued activities in multiple areas of life (socialising, education, and activities). The ability to be flexible and spontaneous without suffering payback was an important theme. However, the concept of recovery was highly individual; there was heterogeneity in the activities children wanted to return to as well as the extent to which different symptoms would be acceptable in recovery. There are differences between child and parent definitions of recovery in CFS/ME, particularly surrounding the role of mood.

Strengths and weaknesses

This is a large qualitative study including children (n=21) and parents (n=22) which reached data saturation (21). As we only interviewed children who were mild/moderately affected, the results are not generalizable to those who are severely affected or younger than 12 years old. We didn't interview children younger than 12 because we did not feel they would be able to answer the questions included in the topic guide. Most children chose to be interviewed without their parents, allowing the exploration of differences between child and parent. In the four interviews where parents were present, the parent was mostly passive, but some contributed a small amount. The child may not have been able to speak freely in these interviews (30). Children were recruited from one specialist service, therefore, the results may not be generalizable to the whole of the UK. Fewer males were

Confidential: For Review Only

recruited (5) and fathers (2) therefore limiting the findings in these sub groups; however, this is consistent with previous research (31).

Results in context with previous literature

Children and parents in this study struggled to define recovery from CFS/ME. This is consistent with the recovery literature in adults with CFS/ME (32). Trouble defining recovery appeared to relate to uncertainty surrounding self-identity; living with the illness had become a ‘new normal’. This reflects the biographical disruption reported in previous research (33). For a lot of children, the focus of recovery had moved away from symptom reduction and towards re-building their lives and identity; a finding that has been reported in the adult CFS/ME population (34).

Parents and children varied in their criteria for recovery, corresponding with the variety of definitions used in the literature (12, 32). Parents suggested a return to functioning at pre-morbid levels or at the level of peers would signify recovery. This is consistent with attempts to characterise recovery as being a return to population norms, equivalent to physician held views (11). Previous research on adult patient’s recovery stories in CFS/ME drew on comparisons between themselves currently and themselves prior to the illness or peers (35). However, ‘pre-morbid’ as a marker is problematic as children in this study varied in their levels of physical function and participation prior to becoming ill, which may change over the course of the illness and memory of pre-morbid levels falls short of an objective measure (36). Similarly, suggestions that recovery would be signified by an increase in activity levels to match those of the children’s peers, reflects the research in the adult

https://mc.manuscriptcentral.com/bmjpo

Confidential: For Review Only

CFS/ME population relating to the normalisation of function. However, this is difficult to define for the same reasons as in the adult population (wide variation for different ages and the presence of co-morbid health problems) (12).

This study identified key areas of life in which recovery would be recognisable (education, socialising, and physical activity). The same areas have been identified in previous research into the development of a patient reported outcome measure (PROM) for use In this population (10). However, this paper highlights that the specific details of what recovery means, is different for each individual. The ability to be spontaneous without suffering payback was an important marker for recovery in this study, further research is needed to understand how children describe and understand payback.

Confidential: For Review Only

IMPLICATIONS

This research highlights the individuality of defining recovery in children with CFS/ME. This suggests that it is vital to involve children in developing any new patient reported outcome measure for paediatric CFS/ME (20). Use of a patient generated index in which patients select the most important areas of their lives and rate their health in those areas may be more appropriate (37). Research studies should consider using questions where patients self-define recovery (e.g. “I have recovered”, or “I have nearly recovered”). Clinicians should be aware of the differences between their own goals for recovery and those of parents and children.

Confidential: For Review Only

ACKNOWLEDGEMENTS

We are grateful to all the children and parents who took part in this study.

FUNDING

This work was supported by a University of Bristol PhD Scholarship. EC was funded by the National Institute for Health Research (Senior Research Fellowship, SRF-2013-06-013). The views expressed in this publication are those of the authors and not necessarily those of the NHS, the National Institute for Health Research or the Department of Health.

COMPETING INTERESTS

All authors declare they have no financial or non-financial interests that may be relevant to the submitted work. EC was the medical advisor for the Association for Young people with ME (AYME) until 2017.

AUTHORS' CONTRIBUTIONS

EC designed the study. RP, NA and DB collected the data. RP and MH analysed the data. All authors contributed to the interpretation of results and to drafting this paper. All authors have read and approved the final version of the manuscript.

DATA SHARING

No additional data available.

ETHICS APPROVAL

Confidential: For Review Only

Full ethical approval was obtained from the NRES Committee North West (08/04/2014, ref 14/NW/0170). R&D approval was obtained from the RNHRD (20/06/2014, ref-RBB 427).

Confidential: For Review Only

What is already known on this topic
• Little is known about how to define recovery in the paediatric CFS/ME population.
**What this study adds**
• Children and their parents struggle to define what would constitute a complete recovery as CFS/ME has become a 'new normal'.
• There is wide variation in definitions of recovery between individuals.
• Recovery definitions go beyond symptom reduction and focus on returning to or achieving the same activity as peers, without payback and with flexibility in routine.

Confidential: For Review Only

References

1. Rimes KA, Goodman R, Hotopf M, Wessely S, Meltzer H, Chalder T. Incidence, prognosis, and risk factors for fatigue and chronic fatigue syndrome in adolescents: a prospective community study. Pediatrics. 2007;119(3):e603-9.
2. Chalder T, Goodman R, Wessely S, Hotopf M, Meltzer H. Epidemiology of chronic fatigue syndrome and self reported myalgic encephalomyelitis in 5-15 year olds: cross sectional study. BMJ. 2003;327(7416):654-5.
3. Crawley EM, Emond AM, Sterne JA. Unidentified Chronic Fatigue Syndrome/myalgic encephalomyelitis (CFS/ME) is a major cause of school absence: surveillance outcomes from school-based clinics. BMJ Open. 2011;1(2):e000252.
4. Crawley E ea. Chronic disabling fatigue at age 13 and association with family adversity. - PubMed - NCBI. 2012.
5. Haines LC, Saidi G, Cooke RW. Prevalence of severe fatigue in primary care. Arch Dis Child. 2005;90(4):367-8.
6. Nijhof SL, Maijer K, Bleijenberg G, Uiterwaal CS, Kimpen JL, van de Putte EM. Adolescent chronic fatigue syndrome: prevalence, incidence, and morbidity. Pediatrics. 2011;127(5):e1169-75.
7. NICE NIfHaCE. Chronic fatigue syndrome/myalgic encephalomyelitis (or encephalopathy): Diagnosis and management of CFS/ME in adults and children: National Institute for Clinical Excellence; 2007.
8. Garralda ME, Rangel L. Impairment and coping in children and adolescents with chronic fatigue syndrome: a comparative study with other paediatric disorders. J Child Psychol Psychiatry. 2004;45(3):543-52.
9. Crawley E, Sterne JA. Association between school absence and physical function in paediatric chronic fatigue syndrome/myalgic encephalopathy. Arch Dis Child. 2009;94(10):752-6.
10. Parslow R, Patel A, Beasant L, Haywood K, Johnson D, Crawley E. What matters to children with CFS/ME? A conceptual model as the first stage in developing a PROM. 2015.
11. Devendorf AR, Jackson CT, Sunnquist M, Jason LA. Defining and measuring recovery from myalgic encephalomyelitis and chronic fatigue syndrome: the physician perspective. Disabil Rehabil. 2017:1-8.
12. Matthees A. Assessment of recovery status in chronic fatigue syndrome using normative data. Quality of Life Research. 2015;24(4):905-7.
13. Nijhof SL, Bleijenberg G, Uiterwaal CS, Kimpen JL, van de Putte EM. Effectiveness of internet-based cognitive behavioural treatment for adolescents with chronic fatigue syndrome (FITNET): a randomised controlled trial. Lancet. 2012;379(9824):1412-8.
14. Brigden A, Parslow RM, Gaunt D, Collin SM, Jones A, Crawley E. Defining the minimally clinically important difference of the SF-36 physical function subscale for paediatric CFS/ME: triangulation using three different methods. Health and quality of life outcomes. 2018;16(1):202.
15. Miller S. Researching children: issues arising from a phenomenological study with children who have diabetes mellitus. J Adv Nurs. 2000;31(5):1228-34.
16. Woodgate RL. Adolescents' perspectives of chronic illness:"it's hard". J Pediatr Nurs. 1998;13(4):210-23.
17. Nicholas DB, Otley A, Smith C, Avolio J, Munk M, Griffiths AM. Challenges and strategies of children and adolescents with inflammatory bowel disease: a qualitative examination. Health and quality of life outcomes. 2007;5(1):28.
18. Ritchie J, Lewis J, Nicholls CM, Ormston R. Qualitative research practice: A guide for social science students and researchers: Sage; 2013.
19. Crawley E, Hunt L, Stallard P. Anxiety in children with CFS/ME. Eur Child Adolesc Psychiatry. 2009;18(11):683-9.

Confidential: For Review Only

20. Matza LS, Patrick DL, Riley AW, Alexander JJ, Rajmil L, Pleil AM, et al. Pediatric patient-reported outcome instruments for research to support medical product labeling: report of the ISPOR PRO good research practices for the assessment of children and adolescents task force. Value Health. 2013;16(4):461-79.
21. Guest G, Bunce A, Johnson L. How many interviews are enough? An experiment with data saturation and variability. Field methods. 2006;18(1):59-82.
22. Castleberry A. NVivo 10 [software program]. Version 10. QSR International; 2012. AJPE; 2014.
23. Braun V, Clarke V. Using thematic analysis in psychology. Research in Psychology. 2006;3(2):77–101.
24. Pope C, Mays N. Qualitative Research in Health care: Blackwell Publishing Ltd; 2006.
25. Pope C, Ziebland S, Mays N. Qualitative research in health care: analysing qualitative data. BMJ: British Medical Journal. 2000;320(7227):114.
26. Britten N. Qualitative interviews in medical research. Bmj. 1995;311(6999):251-3.
27. Johnson RB. Examining the validity structure of qualitative research. Education. 1997;118(2):282.
28. Mays N, Pope C. Assessing quality in qualitative research. BMJ. 2000;320(7226):50-2.
29. Pope C, Mays N. Qualitative research: rigour and qualitative research. Br Med J. 1995;311:109-12.
30. Grills AE, Ollendick TH. Issues in parent-child agreement: the case of structured diagnostic interviews. Clinical child and family psychology review. 2002;5(1):57-83.
31. Missen A, Hollingworth W, Eaton N, Crawley E. The financial and psychological impacts on mothers of children with chronic fatigue syndrome (CFS/ME). Child: care, health and development. 2012;38(4):505-12.
32. Adamowicz JL, Caikauskaite I, Friedberg F. Defining recovery in chronic fatigue syndrome: a critical review. Qual Life Res. 2014;23(9):2407-16.
33. Parslow RM, Harris S, Broughton J, Alattas A, Crawley E, Haywood K, et al. Childrens experiences of chronic fatigue syndrome/myalgic encephalomyelitis (CFS/ME): a systematic review and meta-ethnography of qualitative studies. BMJ open. 2017;7(1):e012633.
34. Whitehead L. Toward a trajectory of identity reconstruction in chronic fatigue syndrome/myalgic encephalomyelitis: A longitudinal qualitative study. International Journal of Nursing Studies. 2006;43(8):1023-31.
35. Brown B, Huszar K, Chapman R. 'Betwixt and between'; liminality in recovery stories from people with myalgic encephalomyelitis (ME) or chronic fatigue syndrome (CFS). Sociology of Health & Illness. 2017.
36. Brooks BL, Kadoura B, Turley B, Crawford S, Mikrogianakis A, Barlow KM. Perception of recovery after pediatric mild traumatic brain injury is influenced by the "good old days" bias: tangible implications for clinical practice and outcomes research. Archives of clinical neuropsychology. 2013;29(2):186-93.
37. Ruta DA, Garratt AM, Leng M, Russell IT, MacDonald LM. A new approach to the measurement of quality of life: the Patient-Generated Index. Med Care. 1994:1109-26.

Confidential: For Review Only

Appendices

Appendix 1: Topic guide

Introduction

1. **How does having CFS/ME affect your life?**

Prompts: Can you tell me more about X, what is it about X, can you describe that…

Recovery

2. **How do you know when you are feeling worse?**
3. **How do you know when you are feeling better?**
4. **What things if any stay the same whether you are better or worse?**

5. **How would you know if you were completely better again?**

Prompts: What would need to improve for you to say that you are better, by how much?

What would you like to do that you are unable to now?

What things can you cope with if they don't improve/ return to normal?

Minimal Clinically Important Difference (MCID)

MCID= change in the measure that a child thinks makes a difference to their lives.

Get YP to fill out SF36 for how they feel now. Take a red pen and get the YP to mark the SMALLEST AMOUNT the responses would have to change to make a difference to them.

6. **What would be the SMALLEST AMOUNT these would have to change by for you to feel like treatment has made a difference to you?**

Prompts: How many responses would have to improve, by how much…

Outcome Domains

Activity: Present YP with outcome domain cards. Add any domains to the blank cards the YP may have spontaneously brought up. Get YP to select the areas most affected by CFS/ME and rank them in order of importance as to what is most important to improve.

Top 5 domains- explore dimensions of domains (frequency/ severity/ duration/ satisfaction)

Prompts e.g. what symptoms bother you most, what is important about school.

[Take a photo of order].

Confidential: For Review Only

7. Pick out the top issues/areas of your life most affected by CFS/ME (upto 10).

8. Imagine you were able to improve these areas, rank these in order of what you feel is most important to improve, put the areas at the top you would most like to improve.

Tiredness

Symptoms (pain, headaches, feeling sick, brain fog)

Sleep problems

Daily activities (getting up, getting dressed, going out)

Payback & crashing (tired after activity)

Fluctuation (changing symptoms- good day vs. bad day)

School (attendance, concentrating, keeping up with work)

Activities & hobbies (sports, clubs)

Spending time with friends

Family activities

Mood (feeling down, worrying)

How you feel about yourself (confidence, personality)

Your future (GCSEs, college, jobs)

Independence (doing things without your parents)

Seeing your boyfriend/girlfriend

9. Why have you ranked them in that order?

Top 5 domains- explore dimensions of domains (frequency/ severity/ duration/ satisfaction)

- Prompts e.g. what symptoms bother you most, what is important about school.

10. How might your answer have been different a year ago? [Pick the cards].

- Why were they important?
- In what ways has it changed/ improved?

<u>Ending question</u>

11. Is there anything else about having CFS/ME that you feel is important to you?

Appendix 2: Additional supporting Quotes

Descriptions of recovery	
(Child-age-12)	"I don't really know, because I've been like this for so long that I don't know when I will be better, because it's not like when I am better it's like

Confidential: For Review Only

	I don't know if I am, because this now is normal for me."
(Parent)	"…I don't think I can honestly answer that. Cos he's been ill for so long."
(Child-age-17)	"I think if I was 100% better than I would be able to do everything that I could do before I actually got it, which was quite a lot."
(Parent)	"…You want to get her to do what other teenagers are doing…"
(Parent)	"so just resuming a bit of normality really. Behaving like a teenager, going into town, you know, seeing friends, that kind of thing."
(Parent)	"…that is one of the most ridiculous questions in the sense that of course she'd know. If you have flu then you know when you're better…"
**Returning to socialising, education, and activities**	
(Child-age-17)	"I wouldn't be missing any days off like college..."
(Child-age-14)	"I'd be able to do more school work, which'd be nice."
(Child-age-17)	"I think a big thing is getting back into exercise, because obviously that was always quite a big part of what I used to….."
(Parent)	"…it would be just being able to yeah, go to school, do a full day…"
(Parent)	"If she started doing some physical exercise…"
(Child-age-15)	"more swimming and go to the gym"
**Recovery as an individualised process**	
(Child-age-17)	"... I would just rather be back how I was, but obviously if there could be like an improvement on one element of it could go, that would be really great. But obviously I would rather go back to how it was before…"
(Child-age-17)	"…I mean I'm the sort of person to just get on with things and achieve what I want to achieve regardless of how I feel, like I don't care if I've got a headache I will go and study and just get the work done. But it's just when it's a combination of being tired, headache, feeling sick, like achy, then it's just like exhausting."
(Parent)	"I don't want her to have any symptoms"
**The absence of 'Pay-Back'**	
(Child-age-13)	"I could do things and I wouldn't get backache"
(Parent)	"…to me he won't be fully recovered until he is like he says walking backwards and forwards to his school, at school all day and coming home, having a snack or whatever, mucking around with his brother,

Confidential: For Review Only

	playing on the computer, and not having the payback afterwards really"
**Freedom and Spontaneity**	
(Child-age-17)	"... and just kind of maybe not having to plan everything quite as much like, because often you know like if we finish at lunchtime, "Oh do you want to go out for lunch?" I'm kind of like, "No I need to go home." But I think yeah just being able to just be like yeah okay I'll come with you, like kind of yeah things like that…"
(Child-age-13)	"…I'd be able to do what I like and do as much as I like and not have to go to bed early, get up early."
(Parent)	"…..I think she'd be able to stay up later at night….."
(Child-age-12)	"walking, erm, walk around for a long amount of time, erm, stand up for a long amount of time"
**Changes in Mood and Motivation**	
(Parent)	"...he would be able to just get up in the morning, and do a full day without any change to his personality or with him feeling anxious…"

Confidential: For Review Only

BMJ Paediatrics Open

PAEDIATRIC CHRONIC FATIGUE SYNDROME PATIENTS' AND PARENTS' PERCEPTIONS OF RECOVERY

Journal:	*BMJ Paediatrics Open*
Manuscript ID	bmjpo-2019-000525.R2
Article Type:	Original research
Date Submitted by the Author:	23-Oct-2019
Complete List of Authors:	Harland, Matthew; University of Bristol, Centre for Academic Child Health<br>Parslow, Roxanne; University of Bristol, Centre for Academic Child Health<br>Anderson, Nina; University of Bristol, Centre for Academic Child Health<br>Byrne, Danielle; University of Bristol, Centre for Academic Child Health<br>Crawley, Esther; University of Bristol, Centre for Academic Child Health
Keywords:	Chronic Fatigue Syndrome, Qualitative research, Patient perspective

SCHOLARONE™
Manuscripts

PAEDIATRIC CHRONIC FATIGUE SYNDROME PATIENTS' AND PARENTS' PERCEPTIONS OF RECOVERY

AUTHOR LIST

Matthew Harland1, Roxanne, M. Parslow1, Nina Anderson1, Danielle Byrne1, Esther Crawley1.

1. Centre for Academic Child Health, Bristol Medical School, University of Bristol, Bristol, England

Corresponding author: Esther Crawley
Centre for Academic Child Health
University of Bristol
Office 1.14
1-5 Whiteladies Road
Bristol
BS8 1NU

+44 (0) 117 42 83073

Email: Esther.Crawley@bristol.ac.uk

Confidential: For Review Only

Key words: Chronic Fatigue Syndrome/Myalgic Encephalomyelitis, Children, Recovery, definitions.

Word count: 2577

(*Excluding title page, abstract, references, figures and tables)*

ABSTRACT

Objectives

Chronic Fatigue Syndrome/Myalgic Encephalomyelitis (CFS/ME) is common in children and adolescents; however, little is known about how we should define recovery. This study aims to explore perceptions of recovery held by paediatric CFS/ME patients and their parents.

Methods

Children with CFS/ME and their parents were recruited through a single specialist paediatric CFS/ME service. Data was collected through semi structured interviews with children and parents. The interview questions explored how participants would know if they/their child had recovered from CFS/ME. Thematic analysis was used to identify patterns within the data.

Results

Twenty-one children with CFS/ME, twenty mothers and two fathers were interviewed. Some children found it hard to define recovery as the illness had become a ‘new normal’. Others thought recovery would indicate returning to pre-morbid levels of activity or achieving the same activity level as peers (socialising, education, and leisure activities). Increased flexibility in routines and the absence of payback after activities were important. The interviews highlighted the concept of recovery as highly individual with wide variation in symptoms experienced, type and level of activity that would signify recovery. Parents describe how changes in mood and motivation would signify their child’s recovery, but children did not reflect on this.

Confidential: For Review Only

Conclusion

Some parents and children struggle to define what would constitute complete recovery. However, signs of recovery were more easily identifiable. Definitions of recovery went far beyond symptom reduction and were focussed towards rebuilding lives.

Confidential: For Review Only

INTRODUCTION

Chronic Fatigue Syndrome/Myalgic Encephalomyelitis (CFS/ME) is common in children and adolescents, with a prevalence of 0.6-2.4% (1-6). It is characterised by severe fatigue and additional symptoms which get worse on exertion including: sleep disturbance, headaches, nausea, musculoskeletal pain, dizziness, and cognitive dysfunction (7). Children with CFS/ME have a reduced quality of life, low school attendance and many develop anxiety and depression (8, 9).

It is difficult to define recovery in CFS/ME as the condition affects multiple dimensions including symptoms, physical activity, social participation (including school), and emotional well-being (10). Improvements can be therefore be measured in physical, psychosocial functioning, and daily activities making it problematic to define recovery in terms of either symptoms or function (11, 12). Some randomised controlled trials have defined recovery as improvement in multiple domains, others as a return to normal within one domain.(13) However, these decisions on defining recovery have been made by researchers and clinicians and not derived from children and young people with CFS/ME. This paper aims to explore how children with CFS/ME and their parents define recovery from the condition. This is important for future research including treatment trials and outcome studies in paediatric CFS/ME.

Confidential: For Review Only

METHOD

Study Design

We recruited participants and their parents/carers to a qualitative study with three research questions: 1. How should we define recovery (this paper); 2. Which outcomes are important for children with CFS/ME and their parents (under review) and 3. What is the minimally clinically important difference in fatigue and SF-36 physical function scale (14). Participants and parents/carers were asked about each of these three areas. The answers to our research question on recovery are reported in this paper.

Interviews were used as it was not possible to recruit to focus groups as patients were from a wide geographical area requiring long distance travel placing a possible burden on children with CFS/ME who experience fatigue and payback. Individual interviews allow more in-depth exploration and interviewing has been used successfully from preschool to high school children with a range of conditions (diabetes, asthma, arthritis and bowel disease (15-17)).

Participants

Children with CFS/ME and their parents were recruited in out-patient clinics provided by a specialist paediatric CFS/ME service. Children were eligible if they were diagnosed with CFS/ME (7), mild to moderately affected (not housebound), aged between 12- 17.99 years and able to understand the patient information sheets. Maximum variation purposive sampling (18) was undertaken to ensure a range of children (age, gender, and disease severity) participated. Children and parents provided the appropriate consent/assent prior

Confidential: For Review Only

to interview. We recruited newly diagnosed patients and patients that were receiving follow up so that a range of views could be included.

Data collection

Semi structured interviews were undertaken by three researchers (RMP, NA, DB) between December 2014 and February 2015. Participants were offered interviews at home or on hospital premises to reduce travel (19). We aimed to interview children and parents separately (20) but families were given the option of being together if they preferred. Interviews took between 15 and 42 minutes. After 20 minutes, the interviewer checked with the participant that they were happy to continue.

A flexible open-ended interview guide was developed with input from a young person's advisory group within a secondary school (7 female, 1 male) to ensure the questions were comprehensible and clear to school aged children. The topic guide included questions on recovery, relevant outcomes to measure, and the amount of change considered important (the minimal clinically important difference). This section included questions on how participants knew when they were feeling worse, better, and how they would know if they had recovered ***(Appendix 1***).

Interviews continued until data saturation was achieved (21).

Data analysis

All interviews were recorded on an encrypted digital audio recorder and transcribed verbatim. Transcripts were checked for accuracy, anonymised and uploaded to NVIVO 10 (22) to provide an audit trail for data analysis. Thematic analysis (23) was used to identify

Confidential: For Review Only

patterns (themes) within the data in an iterative process. Codes were initially developed "in vivo" inductively from participants own language in order to represent concepts important to families with CFS/ME. Codes were then added, merged, or split as analysis progressed to reflect emerging dimensions in the data (24). This allowed higher-level recurring themes to be developed and the data was checked between participants to see if matched the overall coding. A summary was then written with illustrative quotes to highlight the overall themes and differing perspectives (25).

Quality assurance

The first three interview transcripts were reviewed to monitor the proficiency of the interviewers (RP, NA, DB) in following the topic guide, picking up on cues, and avoiding leading questions (26). The transcripts were then discussed in a meeting with feedback from a senior researcher (EC). The interview transcripts were double coded and themes discussed between researchers (MH, RP) to compare coding, highlight any significant differences to improve the trustworthiness of analyses and enhance interpretation (27-29). Coding was found to be very similar, at times alternative wording was used for the same codes.

Patient and public involvement

A young person's advisory group comprising secondary school children was involved in the development of the interview topic guide during the design stages of this study.

Confidential: For Review Only

RESULTS

Participants

Twenty-one children with CFS/ME were interviewed, 16 (76%) were female and 5 (24%) were male, mean age was 14.4 years and ages ranged from 12 to 17 years old. Seventeen (81%) children had mild CFS/ME and 4 (19%) had moderate CFS/ME. Twenty mothers and two fathers were interviewed. Most children and parents were interviewed separately but in 4/21 interviews a parent was present in the room during the child's interview. Five families who had initially expressed interest in participating when being contacted by the researcher did not participate (two were too busy and three children were too ill).

Difficulty defining recovery

Some children found it difficult to describe how they would know if they had recovered. Living with the condition had become a 'new normal' for them and as they had had CFS/ME for so long, children and their parents couldn't imagine recovery. Due to the fluctuating nature of the illness one older child felt that even if she had recovered she would worry about relapsing:

> "I don't really think I would know. I think like now it's constantly at the back of mind like whether or not like it will knock me again..." (Child-age-17)

Parents and children both had a tendency to report signs of improvement rather than signs of complete recovery. Some children, but more parents, stated that they would have no difficulty in knowing when they/their child had recovered as they would be 'back-to-normal' engaging in pre-morbid levels of functioning or the same activity as their peers ***(additional***

Confidential: For Review Only

quotes supporting each theme can be found in Appendix 2):

> "I think we would know because she'd be back to how she was before sort of the illness …You know, there's that reference point in time that you can go back to." (Parent)

Returning to socialising, education, and activities

All children reported that recovery would be indicated by a return to activities that were particularly important to them: socialising with friends, attending school/college regularly, and both physical "more swimming and go to the gym" and mental activities "I think I'd just have a bit better memory" (Child-age-15). Often, children described school attendance as important for social not educational reasons. The same categories were also reported as important indicators of recovery by parents.

> "Probably school, I'd say – - 'cause I think, if I get back into school then I can like, get in touch with more people" (Child-age-13)

Being able to return to activities but do them for longer was important "walking, erm, walk around for a long amount of time, erm, stand up for a long amount of time" (Child-age-12).

Recovery as an individualised process

There was wide variation in types of activities that children and parents described as indicating recovery. For children, these activities included cooking, playing instruments, singing, and creating artwork. The types of activities children referred to were relevant for their age. For example, 16-17 year olds talked about wanting to be able to be more independent and "walk to town", whereas younger children talked about "sleepovers" and playing with friends, "run around and play a lot".

Confidential: For Review Only

"…and having more independence in that sense. Like most people my age will go out to the shops, or just walk around aimlessly, that seems to be popular, but like [laughs] I wish I could do that like. (Child-age-16)

"Erm, I miss my sports. Erm, ju- have, have sleepovers, erm, with my friends" (Child-age-12)

For parents, these included activities with the family, travel, holidays, and dog-walks. For one family, both the parent and child described increased mobility to be the most significant indicator of recovery as the child was reliant on a wheelchair.

"...knows what she wants to do, if she could do it she would, and her main aim is now to probably try and get round the shops without her wheelchair…" (Parent)

In contrast, other parents described a much higher levels of activity to signify recovery:

"…on the days when gymnastics was on, she exercised every single day, and that's how I know when I think when she's better it's when she starts physical activity again." (Parent)

The idea that recovery is highly individualised was also evident in the descriptions of symptoms children could tolerate as part of their recovery. Some parents and children stated that no symptoms would be acceptable in recovery. However, some children felt that they would be able to consider themselves recovered even if they still had some symptoms. Children had different views over whether tiredness was bearable or not and which symptoms were tolerable.

"I'd be able to live with my headaches." (Child-age-14)

"…the headaches because they, erm, they just stop her being able to think…" (Parent)

Confidential: For Review Only

The absence of 'Payback'

As well as returning to premorbid functioning, children and parents felt that increasing activities should not result in payback (the increase in symptoms following activity) in order for them to be considered recovered.

"I think I'd know if like, I went out and did something, I didn't get payback for it, I think that one, I'd know that I was like really starting to recover" (Child-age-13)

Freedom and Spontaneity

Older children talked about how they had become used to planning their activity in a way that facilitated pacing and reduced payback. They felt that life had become regimented and that they were unable to spontaneously choose to do an activity/continue without a break. The absence of this rigidity of routine was an important indicator of recovery.

"…like won't do as much during the day so that then I can go out in the evening. Yeah, I think that's like that what I mean by like planning, it's just so I can kind of plan my other activities like accordingly I suppose. (Child-age-17).

"You'd know when she's recovered because she'd be back to normal, because she'd be able to do things, and not just do them but do them without thinking about how much energy she was using." (Parent)

For some parents and children, recovery also incorporated the idea that they would be able to have more flexibility in their sleep routines, without experiencing payback. In contrast, some parents felt that the monitoring of activity would be acceptable in recovery to help limit tiredness.

Confidential: For Review Only

Changes in Mood and Motivation

Parents expressed that recovery would be indicated by a change in their child's mood. For some, this would be a reduction in anxiety, and for others, it would be increased positive mood and more engagement with others:

"Well if she was completely better she would be lively again, bubbly again, joking and laughing again, singing again, but all the time, that's how she would be." (Parent)

Often, parents described that their children would be more motivated when they had recovered. This position was not reflected in the responses of children who often appeared motivated but cautious and limited by their symptoms.

"So if he was better you'd think he'd just be motivated to get up and be physically able." (Parent)

Some parents recognised that changes in personality and preference in activities in their children may be due to factors other than CFS/ME.

"Yeah, she's hit puberty as well so, you know, I think a lot of the sort of withdrawing kind of is a teenage thing as well, like wanting to be on her own." (Parent)

Confidential: For Review Only

DISCUSSION

This is the first paper to explore what children with CFS/ME feel about recovery. There is a struggle to define what would constitute complete recovery as CFS/ME has become a 'new normal'. Previous levels of activity prior to becoming ill or keeping up with peers were key markers. Descriptions of recovery went far beyond symptom reduction and focussed on rebuilding lives which incorporated valued activities in multiple areas of life (socialising, education, and activities). The ability to be flexible and spontaneous without suffering payback was an important theme. However, the concept of recovery was highly individual; there was heterogeneity in the activities children wanted to return to as well as the extent to which different symptoms would be acceptable in recovery. There are differences between child and parent definitions of recovery in CFS/ME, particularly surrounding the role of mood.

Strengths and weaknesses

This is a large qualitative study including children (n=21) and parents (n=22) which reached data saturation. We considered we had reached data saturation when no new themes arose from the data (21). As we only interviewed children who were mild/moderately affected, the results are not generalizable to those who are severely affected or younger than 12 years old. We didn't interview children younger than 12 because we did not feel they would be able to answer the questions included in the topic guide. Most children chose to be interviewed without their parents, allowing the exploration of differences between child and parent. In the four interviews where parents were present, the parent was mostly passive, but some contributed a small amount. The child may not have been able to speak freely in these interviews (30). Children were recruited from one specialist service,

https://mc.manuscriptcentral.com/bmjpo

Confidential: For Review Only

therefore, the results may not be generalizable to the whole of the UK. Fewer males were recruited (5) and fathers (2) therefore limiting the findings in these sub groups; however, this is consistent with previous research (31).

Results in context with previous literature

Children and parents in this study struggled to define recovery from CFS/ME. This is consistent with the recovery literature in adults with CFS/ME (32). Trouble defining recovery appeared to relate to uncertainty surrounding self-identity; living with the illness had become a 'new normal'. This reflects the biographical disruption reported in previous research (33). For a lot of children, the focus of recovery had moved away from symptom reduction and towards re-building their lives and identity; a finding that has been reported in the adult CFS/ME population (34).

Parents and children varied in their criteria for recovery, corresponding with the variety of definitions used in the literature (12, 32). Parents suggested a return to functioning at pre-morbid levels or at the level of peers would signify recovery. This is consistent with attempts to characterise recovery as being a return to population norms, equivalent to physician held views (11). Previous research on adult patient's recovery stories in CFS/ME drew on comparisons between themselves currently and themselves prior to the illness or peers (35). However, 'pre-morbid' as a marker is problematic as children in this study varied in their levels of physical function and participation prior to becoming ill, which may change over the course of the illness and memory of pre-morbid levels falls short of an objective measure (36). Similarly, suggestions that recovery would be signified by an increase in

Confidential: For Review Only

activity levels to match those of the children's peers, reflects the research in the adult CFS/ME population relating to the normalisation of function. However, this is difficult to define for the same reasons as in the adult population (wide variation for different ages and the presence of co-morbid health problems) (12).

This study identified key areas of life in which recovery would be recognisable (education, socialising, and physical activity). The same areas have been identified in previous research into the development of a patient reported outcome measure (PROM) for use In this population (10). However, this paper highlights that the specific details of what recovery means, is different for each individual. The ability to be spontaneous without suffering payback was an important marker for recovery in this study, further research is needed to understand how children describe and understand payback.

Confidential: For Review Only

Confidential: For Review Only

IMPLICATIONS

This research highlights the individuality of defining recovery in children with CFS/ME. This suggests that it is vital to involve children in developing any new patient reported outcome measure for paediatric CFS/ME (20). Use of a patient generated index in which patients select the most important areas of their lives and rate their health in those areas may be more appropriate (37). Research studies should consider using questions where patients self-define recovery (e.g. “I have recovered”, or “I have nearly recovered”). Clinicians should be aware of the differences between their own goals for recovery and those of parents and children.

ACKNOWLEDGEMENTS

We are grateful to all the children and parents who took part in this study.

FUNDING

This work was supported by a University of Bristol PhD Scholarship. EC was funded by the National Institute for Health Research (Senior Research Fellowship, SRF-2013-06-013). The views expressed in this publication are those of the authors and not necessarily those of the NHS, the National Institute for Health Research or the Department of Health.

COMPETING INTERESTS

All authors declare they have no financial or non-financial interests that may be relevant to the submitted work. EC was the medical advisor for the Association for Young people with ME (AYME) until 2017.

AUTHORS' CONTRIBUTIONS

EC designed the study. RP, NA and DB collected the data. RP and MH analysed the data. All authors contributed to the interpretation of results and to drafting this paper. All authors have read and approved the final version of the manuscript.

DATA SHARING

No additional data available.

ETHICS APPROVAL

Confidential: For Review Only

Full ethical approval was obtained from the NRES Committee North West (08/04/2014, ref 14/NW/0170). R&D approval was obtained from the RNHRD (20/06/2014, ref-RBB 427).

Confidential: For Review Only

What is already known on this topic
• Little is known about how to define recovery in the paediatric CFS/ME population.
**What this study adds**
• Children and their parents struggle to define what would constitute a complete recovery as CFS/ME has become a 'new normal'.
• There is wide variation in definitions of recovery between individuals.
• Recovery definitions go beyond symptom reduction and focus on returning to or achieving the same activity as peers, without payback and with flexibility in routine.

Confidential: For Review Only

References

1. Rimes KA, Goodman R, Hotopf M, Wessely S, Meltzer H, Chalder T. Incidence, prognosis, and risk factors for fatigue and chronic fatigue syndrome in adolescents: a prospective community study. Pediatrics. 2007;119(3):e603-9.
2. Chalder T, Goodman R, Wessely S, Hotopf M, Meltzer H. Epidemiology of chronic fatigue syndrome and self reported myalgic encephalomyelitis in 5-15 year olds: cross sectional study. BMJ. 2003;327(7416):654-5.
3. Crawley EM, Emond AM, Sterne JA. Unidentified Chronic Fatigue Syndrome/myalgic encephalomyelitis (CFS/ME) is a major cause of school absence: surveillance outcomes from school-based clinics. BMJ Open. 2011;1(2):e000252.
4. Crawley E ea. Chronic disabling fatigue at age 13 and association with family adversity. - PubMed - NCBI. 2012.
5. Haines LC, Saidi G, Cooke RW. Prevalence of severe fatigue in primary care. Arch Dis Child. 2005;90(4):367-8.
6. Nijhof SL, Maijer K, Bleijenberg G, Uiterwaal CS, Kimpen JL, van de Putte EM. Adolescent chronic fatigue syndrome: prevalence, incidence, and morbidity. Pediatrics. 2011;127(5):e1169-75.
7. NICE NIfHaCE. Chronic fatigue syndrome/myalgic encephalomyelitis (or encephalopathy): Diagnosis and management of CFS/ME in adults and children: National Institute for Clinical Excellence; 2007.
8. Garralda ME, Rangel L. Impairment and coping in children and adolescents with chronic fatigue syndrome: a comparative study with other paediatric disorders. J Child Psychol Psychiatry. 2004;45(3):543-52.
9. Crawley E, Sterne JA. Association between school absence and physical function in paediatric chronic fatigue syndrome/myalgic encephalopathy. Arch Dis Child. 2009;94(10):752-6.
10. Parslow R, Patel A, Beasant L, Haywood K, Johnson D, Crawley E. What matters to children with CFS/ME? A conceptual model as the first stage in developing a PROM. 2015.
11. Devendorf AR, Jackson CT, Sunnquist M, Jason LA. Defining and measuring recovery from myalgic encephalomyelitis and chronic fatigue syndrome: the physician perspective. Disabil Rehabil. 2017:1-8.
12. Matthees A. Assessment of recovery status in chronic fatigue syndrome using normative data. Quality of Life Research. 2015;24(4):905-7.
13. Nijhof SL, Bleijenberg G, Uiterwaal CS, Kimpen JL, van de Putte EM. Effectiveness of internet-based cognitive behavioural treatment for adolescents with chronic fatigue syndrome (FITNET): a randomised controlled trial. Lancet. 2012;379(9824):1412-8.
14. Brigden A, Parslow RM, Gaunt D, Collin SM, Jones A, Crawley E. Defining the minimally clinically important difference of the SF-36 physical function subscale for paediatric CFS/ME: triangulation using three different methods. Health and quality of life outcomes. 2018;16(1):202.
15. Miller S. Researching children: issues arising from a phenomenological study with children who have diabetes mellitus. J Adv Nurs. 2000;31(5):1228-34.
16. Woodgate RL. Adolescents' perspectives of chronic illness:"it's hard". J Pediatr Nurs. 1998;13(4):210-23.

Confidential: For Review Only

17. Nicholas DB, Otley A, Smith C, Avolio J, Munk M, Griffiths AM. Challenges and strategies of children and adolescents with inflammatory bowel disease: a qualitative examination. Health and quality of life outcomes. 2007;5(1):28.
18. Ritchie J, Lewis J, Nicholls CM, Ormston R. Qualitative research practice: A guide for social science students and researchers: Sage; 2013.
19. Crawley E, Hunt L, Stallard P. Anxiety in children with CFS/ME. Eur Child Adolesc Psychiatry. 2009;18(11):683-9.
20. Matza LS, Patrick DL, Riley AW, Alexander JJ, Rajmil L, Pleil AM, et al. Pediatric patient-reported outcome instruments for research to support medical product labeling: report of the ISPOR PRO good research practices for the assessment of children and adolescents task force. Value Health. 2013;16(4):461-79.
21. Guest G, Bunce A, Johnson L. How many interviews are enough? An experiment with data saturation and variability. Field methods. 2006;18(1):59-82.
22. Castleberry A. NVivo 10 [software program]. Version 10. QSR International; 2012. AJPE; 2014.
23. Braun V, Clarke V. Using thematic analysis in psychology. Research in Psychology. 2006;3(2):77–101.
24. Pope C, Mays N. Qualitative Research in Health care: Blackwell Publishing Ltd; 2006.
25. Pope C, Ziebland S, Mays N. Qualitative research in health care: analysing qualitative data. BMJ: British Medical Journal. 2000;320(7227):114.
26. Britten N. Qualitative interviews in medical research. Bmj. 1995;311(6999):251-3.
27. Johnson RB. Examining the validity structure of qualitative research. Education. 1997;118(2):282.
28. Mays N, Pope C. Assessing quality in qualitative research. BMJ. 2000;320(7226):50-2.
29. Pope C, Mays N. Qualitative research: rigour and qualitative research. Br Med J. 1995;311:109-12.
30. Grills AE, Ollendick TH. Issues in parent-child agreement: the case of structured diagnostic interviews. Clinical child and family psychology review. 2002;5(1):57-83.
31. Missen A, Hollingworth W, Eaton N, Crawley E. The financial and psychological impacts on mothers of children with chronic fatigue syndrome (CFS/ME). Child: care, health and development. 2012;38(4):505-12.
32. Adamowicz JL, Caikauskaite I, Friedberg F. Defining recovery in chronic fatigue syndrome: a critical review. Qual Life Res. 2014;23(9):2407-16.
33. Parslow RM, Harris S, Broughton J, Alattas A, Crawley E, Haywood K, et al. Childrens experiences of chronic fatigue syndrome/myalgic encephalomyelitis (CFS/ME): a systematic review and meta-ethnography of qualitative studies. BMJ open. 2017;7(1):e012633.
34. Whitehead L. Toward a trajectory of identity reconstruction in chronic fatigue syndrome/myalgic encephalomyelitis: A longitudinal qualitative study. International Journal of Nursing Studies. 2006;43(8):1023-31.
35. Brown B, Huszar K, Chapman R. 'Betwixt and between'; liminality in recovery stories from people with myalgic encephalomyelitis (ME) or chronic fatigue syndrome (CFS). Sociology of Health & Illness. 2017.
36. Brooks BL, Kadoura B, Turley B, Crawford S, Mikrogianakis A, Barlow KM. Perception of recovery after pediatric mild traumatic brain injury is influenced by the "good old days" bias: tangible implications for clinical practice and outcomes research. Archives of clinical neuropsychology. 2013;29(2):186-93.

Confidential: For Review Only

37. Ruta DA, Garratt AM, Leng M, Russell IT, MacDonald LM. A new approach to the measurement of quality of life: the Patient-Generated Index. Med Care. 1994:1109-26.

Confidential: For Review Only

Appendices

Appendix 1: Topic guide

Introduction

1. **How does having CFS/ME affect your life?**

Prompts: Can you tell me more about X, what is it about X, can you describe that…

Recovery

2. **How do you know when you are feeling worse?**
3. **How do you know when you are feeling better?**
4. **What things if any stay the same whether you are better or worse?**

5. **How would you know if you were completely better again?**

Prompts: What would need to improve for you to say that you are better, by how much?

What would you like to do that you are unable to now?

What things can you cope with if they don't improve/ return to normal?

Minimal Clinically Important Difference (MCID)

MCID= change in the measure that a child thinks makes a difference to their lives.

Get YP to fill out SF36 for how they feel now. Take a red pen and get the YP to mark the SMALLEST AMOUNT the responses would have to change to make a difference to them.

6. **What would be the SMALLEST AMOUNT these would have to change by for you to feel like treatment has made a difference to you?**

Prompts: How many responses would have to improve, by how much…

Outcome Domains

Activity: Present YP with outcome domain cards. Add any domains to the blank cards the YP may have spontaneously brought up. Get YP to select the areas most affected by CFS/ME and rank them in order of importance as to what is most important to improve.

Top 5 domains- explore dimensions of domains (frequency/ severity/ duration/ satisfaction)

Prompts e.g. what symptoms bother you most, what is important about school.

[Take a photo of order].

Confidential: For Review Only

7. Pick out the top issues/areas of your life most affected by CFS/ME (upto 10).

8. Imagine you were able to improve these areas, rank these in order of what you feel is most important to improve, put the areas at the top you would most like to improve.

Tiredness

Symptoms (pain, headaches, feeling sick, brain fog)

Sleep problems

Daily activities (getting up, getting dressed, going out)

Payback & crashing (tired after activity)

Fluctuation (changing symptoms- good day vs. bad day)

School (attendance, concentrating, keeping up with work)

Activities & hobbies (sports, clubs)

Spending time with friends

Family activities

Mood (feeling down, worrying)

How you feel about yourself (confidence, personality)

Your future (GCSEs, college, jobs)

Independence (doing things without your parents)

Seeing your boyfriend/girlfriend

9. Why have you ranked them in that order?

Top 5 domains- explore dimensions of domains (frequency/ severity/ duration/ satisfaction)

- Prompts e.g. what symptoms bother you most, what is important about school.

10. How might your answer have been different a year ago? [Pick the cards].

- Why were they important?
- In what ways has it changed/ improved?

Ending question

11. Is there anything else about having CFS/ME that you feel is important to you?

Appendix 2: Additional supporting Quotes

Descriptions of recovery	
(Child-age-12)	"I don't really know, because I've been like this for so long that I don't know when I will be better, because it's not like when I am better it's like

Confidential: For Review Only

	I don't know if I am, because this now is normal for me."
(Parent)	"…I don't think I can honestly answer that. Cos he's been ill for so long."
(Child-age-17)	"I think if I was 100% better than I would be able to do everything that I could do before I actually got it, which was quite a lot."
(Parent)	"…You want to get her to do what other teenagers are doing…"
(Parent)	"so just resuming a bit of normality really. Behaving like a teenager, going into town, you know, seeing friends, that kind of thing."
(Parent)	"…that is one of the most ridiculous questions in the sense that of course she'd know. If you have flu then you know when you're better…"
**Returning to socialising, education, and activities**	
(Child-age-17)	"I wouldn't be missing any days off like college..."
(Child-age-14)	"I'd be able to do more school work, which'd be nice."
(Child-age-17)	"I think a big thing is getting back into exercise, because obviously that was always quite a big part of what I used to….."
(Parent)	"…it would be just being able to yeah, go to school, do a full day…"
(Parent)	"If she started doing some physical exercise…"
(Child-age-15)	"more swimming and go to the gym"
**Recovery as an individualised process**	
(Child-age-17)	"... I would just rather be back how I was, but obviously if there could be like an improvement on one element of it could go, that would be really great. But obviously I would rather go back to how it was before…"
(Child-age-17)	"…I mean I'm the sort of person to just get on with things and achieve what I want to achieve regardless of how I feel, like I don't care if I've got a headache I will go and study and just get the work done. But it's just when it's a combination of being tired, headache, feeling sick, like achy, then it's just like exhausting."
(Parent)	"I don't want her to have any symptoms"
**The absence of 'Pay-Back'**	
(Child-age-13)	"I could do things and I wouldn't get backache"
(Parent)	"…to me he won't be fully recovered until he is like he says walking backwards and forwards to his school, at school all day and coming home, having a snack or whatever, mucking around with his brother,

Confidential: For Review Only

	playing on the computer, and not having the payback afterwards really”
**Freedom and Spontaneity**	
(Child-age-17)	“... and just kind of maybe not having to plan everything quite as much like, because often you know like if we finish at lunchtime, “Oh do you want to go out for lunch?” I’m kind of like, “No I need to go home.” But I think yeah just being able to just be like yeah okay I’ll come with you, like kind of yeah things like that…”
(Child-age-13)	“…I'd be able to do what I like and do as much as I like and not have to go to bed early, get up early.”
(Parent)	“…..I think she’d be able to stay up later at night…..”
(Child-age-12)	“walking, erm, walk around for a long amount of time, erm, stand up for a long amount of time”
**Changes in Mood and Motivation**	
(Parent)	“...he would be able to just get up in the morning, and do a full day without any change to his personality or with him feeling anxious…”

Confidential: For Review Only
